# The Xenoreactome: Where Foreign Chemistry Becomes Biological Organization

**DOI:** 10.1021/jacsau.6c01406

**Published:** 2026-10-14

**Authors:** Andrea Belluati

**Affiliations:** † Max Planck Institute for Polymer Research, Ackermannweg 10, 55128 Mainz, Germany; ‡ Department of Chemistry, Technical University of Darmstadt, Peter-Grünberg-Straße 4, 64287 Darmstadt, Germany; § Centre for Synthetic Biology, Technical University of Darmstadt, Merckstraße 25, 64283 Darmstadt, Germany

**Keywords:** xenoreactome, new-to-nature chemistry, synthetic biology, biocatalysis, artificial cells, cellular polymerization, metabolic engineering, biohybrid materials

## Abstract

Biological systems can perform chemistry beyond their evolved reaction networks, but a reaction that is chemically possible does not necessarily occur in an intact system. I define the xenoreactome as the state- and reference-dependent network through which foreign chemistry enters a biological or cell-like system, is transformed and propagated, and is ultimately cleared or retained. The concept focuses on the factors that determine whether foreign chemistry is realized, including transport, localization, physiological state, downstream transfer, and clearance. Xenoreactomes may remain entirely molecular, carry non-native chemical or elemental features through existing biological machinery, or support reaction sequences with no direct biological counterpart, such as sustained free-radical vinyl polymerization. In some cases, xenochemical products can also accumulate sufficiently to form polymers, condensed phases, or other material states. For these systems, I introduce a simple quantitative criterion that relates xenochemical production and persistence to the threshold required for material formation. The xenoreactome provides a framework for asking which foreign chemistry biological systems can actually support, how far it propagates, and what ultimately happens to its products.

## Graphical abstract

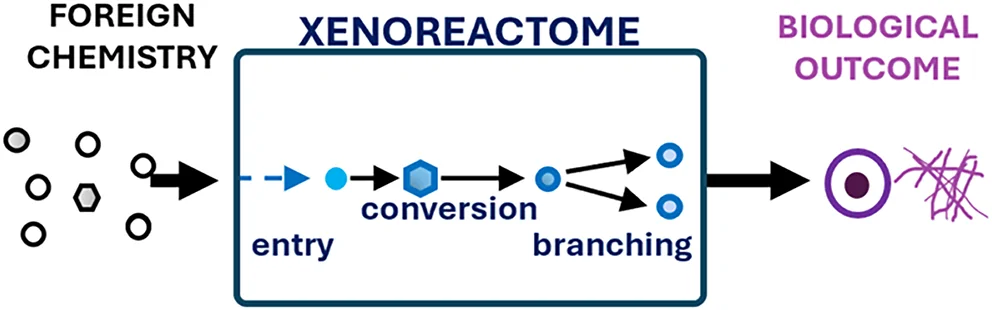

## Chemistry Beyond the Evolved Reaction Network

Every cell is, to some extent, a chemical stranger to itself.

Enzymes recognize imperfectly,^1^ metabolites undergo spontaneous damage,^2^ and physical organization continually exposes or suppresses chemical possibilities beyond annotated reaction networks. Living systems also encounter molecules from other organisms, geochemical processes, combustion and degradation, as well as pharmaceuticals, pesticides and industrial compounds that appeared only recently on evolutionary time scales. Taken together, this means that biology operates in a chemical environment broader than its conventionally annotated pathways. Engineered systems now make the same problem deliberate. Artificial metalloenzymes, engineered fluorination pathways, bioorthogonal reaction networks (designed to operate selectively in the presence of native cellular chemistry) and cellular radical polymerizations extend the chemical repertoire of living or biomimetic compartments beyond ordinary biosynthesis.^3–5^ Facing these chemical possibilities, what can an organism actually realize?

Biology is already full of partial answers. Starting points for new functions can arise from enzyme promiscuity;^1,6^ underground metabolism can connect side activities into condition-dependent pathways;^7,8^ and metabolite-damage research shows how aberrant products are generated, repaired or pre-empted.^2^ These fields have established that physiological reaction networks are chemically permeable and that reactions outside canonical metabolism are an ordinary feature of cellular chemistry. A complementary architectural view emphasizes that metabolic function depends not only on which reactions are present, but on how they are connected, spatially organized and temporally constrained. The same biochemical components can therefore support different functional regimes depending on routing and context.^9^

Xenobiotic metabolism already encompasses the transformation, transport, compartmentalization and excretion of foreign compounds, while the xenometabolome describes the molecular products that result.^10^ The micrometabolism hypothesis further emphasizes that xenobiotic fate reflects the reactions permitted by the available enzymes, their affinities and the chemistry of the substrate.^11^ Absorption, distribution, metabolism and excretion (ADME) organize these processes around the fate of a xenobiotic after exposure. Rather than following only the fate of an encountered xenobiotic, the xenoreactome asks whether foreign chemistry – including chemistry deliberately generated within the system – is realized as connected transformations and transfers. This adds a constructive question that is not the usual starting point of xenobiotic metabolism: what foreign chemistry can a biological or cell-like system be engineered to realize? The problem is therefore no longer whether a reaction is possible, but whether the system allows it to become connected chemistry.

Chemical possibility is a surprisingly poor predictor of what a living system will actually do. A catalyst may be present without gaining access to its substrate; a xenometabolite may be detected without participating in a downstream cascade. Reactions readily observed in purified systems can disappear in cells through exclusion, competition or quenching, whereas weak reactions can become consequential through confinement, accumulation, or slow clearance. Access, localization, transfer and persistence therefore determine how far foreign chemistry propagates and where it terminates.

The xenoreactome describes this intermediate level between molecular possibility and biological outcome by considering chemical reactivity together with transport, compartmentalization and clearance. Engineered reaction networks and molecular-to-material transitions can be considered in the same way when their realization depends on biological organization.

## Defining the Xenoreactome

The xenoreactome is defined here as:

*The state- and reference-dependent network through which foreign chemistry enters a biological or cell-like system, is transformed and propagated, and is ultimately cleared or retained.*

Here, state refers to the condition of the biological or cell-like system at the time of observation, whereas reference denotes the declared baseline against which chemistry is judged foreign. A xenoreactome is therefore not a catalogue of reactions. What it contains depends on the system being considered, its physiological state, its spatial organization and the time scale of observation. A useful representation can treat chemical species in defined compartments or physical states as nodes, and transformations or transfers between them as edges. Clearance and retention then mark possible end points. Here, network does not imply a large metabolic pathway or a minimum number of chemical transformations, and a single dominant xenoreaction can be considered together with the access, localization and loss processes that determine its realization and fate. The defining feature is the dependence of the observed chemistry on the organization of the system, rather than the number of catalytic steps.

But what counts as foreign? It does not simply mean synthetic or anthropogenic, nor does foreign imply toxic, beneficial or evolutionarily unprecedented: these are separate properties of the chemistry and its consequences. A molecule may be part of the normal chemistry of one organism and foreign to another. Fluoroacetate, for example, occurs naturally in some plants but is foreign to mammalian metabolism.^12^ The reference point should therefore be stated explicitly -for example, the evolved chemical repertoire of an unmodified host, the current genotype or a defined community- and this boundary can shift as a system evolves or is engineered. Genetic encoding alone does not necessarily erase foreignness if the declared comparison is to the evolved chemical repertoire of the host.

Xenobiology provides the broader program of constructing and studying biological systems whose molecular building blocks, metabolism, information processing or chemical dependencies depart from evolved biology. The xenoreactome addresses a complementary analytical question: within a defined biological or cell-like system, which chemistry foreign to the chosen reference state is actually realized, how is it propagated, and where does it terminate? Xenobiological engineering changes the repertoire and architecture of a system; xenoreactomic analysis follows the transformations and transfers that this architecture actually supports under specified conditions. The two perspectives therefore overlap without being interchangeable: an engineered molecular component establishes a chemical possibility, whereas its realized routing and fate must be determined in the organized system.^13–16^ It also occupies a different analytical level from the exposome and xenometabolome: the exposome describes what a system encounters,^17^ the xenometabolome the molecules that result,^10^ and the xenoreactome the reactions and transfers through which one becomes the other (Figure 1A).

**Figure 1:**
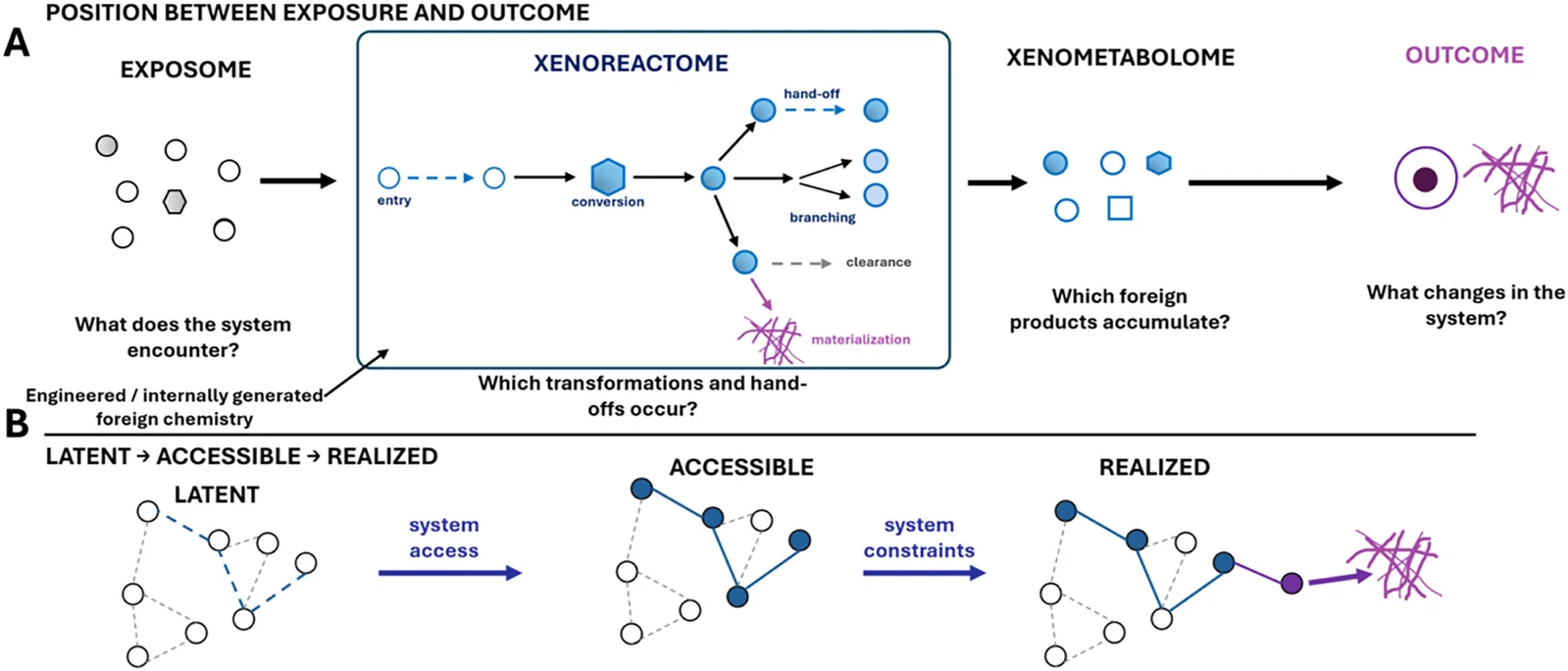
Analytical position of the xenoreactome. (A) The exposome defines chemical inputs encountered by a reference system, the xenoreactome describes the transformations and physical or catalytic hand-offs that determine their fate, and the xenometabolome records foreign-derived molecules detected as outputs. (B) The lower logic emphasizes chemical possibility → system access → observed flux, with transport, localization, state and clearance determining realization.

A single promiscuous enzyme–substrate interaction represents only one possible reaction. System organization matters when it determines which foreign reactions occur and how far they proceed. Entry and localization determine which reactants can meet; transfer between catalysts or compartments determines whether one reaction feeds the next; clearance and persistence determine whether products disappear, propagate or accumulate. Material formation is a further consequence in systems that cross a physical threshold.

An isolated reaction in a purified system is therefore a xenoreaction, but not a xenoreactome. Nor does a collection of foreign-derived metabolites become a xenoreactome simply because several of them can be detected. If system organization does not affect access, propagation or fate, xenobiotic metabolism or conventional reaction chemistry is sufficient. A foreign reaction can occur in an organized biological system without benefiting the host, being tolerated or becoming stable over generations. Physiological accommodation, functional integration and evolutionary stabilization are separate outcomes that require their own evidence. The xenoreactome describes the chemistry that actually occurs, how it propagates and what happens to its products. It therefore includes toxic conversions, transient reactions and products that are ultimately cleared.

The organization that shapes this chemistry need not be enzymatic or membrane-bound. Cell-like systems include biomimetic assemblies in which partitioning, exchange or retention affects reactions. A coacervate (a membraneless phase-separated compartment) can therefore support a xenoreactome if it changes substrate access, reaction propagation or product fate but compartmentalization alone is insufficient. Membranes, compartments and biological materials can influence local concentrations, redox conditions, precursor supply, catalyst assembly and transfer between reaction sites, even when individual reactions occur spontaneously.

The same criterion applies to biotechnology. A purified enzyme converting a substrate establishes a reaction, but not by itself a xenoreactome. Whole-cell biotransformations fall within the framework when uptake, cofactor supply, competing metabolism, export or retention shapes the conversion and its products. Xenoreactomic analysis asks how these features enable or constrain foreign chemistry. This leads to a practical set of experimental questions for identifying and comparing xenoreactomes (Table 1).

**Table 1:** Experimental Questions for Identifying and Comparing Xenoreactomes

question	what to determine	representative evidence
Can the chemistry occur?	catalytic or spontaneous capacity under chemically permissive conditions.	purified catalyst, lysate or defined in vitro reaction; product identity.
Can substrate and catalyst meet?	entry, permeability, partitioning, localization and cofactor availability.	transport perturbation, compartment-specific sampling, localization or partition measurements.
Does the chemistry propagate?	downstream transformations and physical hand-offs across catalysts, compartments or organisms.	time-resolved intermediates, isotope tracing, knockout/inhibitor interruption of a required hand-off.
How long does the product persist?	competition between production and consumption, repair, export, dilution and physical loss.	time-resolved flux and clearance measurements; effective pretransition persistence time, τ_eff_.
Does foreign chemistry become retained?	persistent residence in a biomolecule, interface or organized structure.	direct localization of the foreign feature or element in the resulting product or structure.
Can a physical threshold be crossed?	production and persistence relative to an operational materialization threshold *X*_crit_ when one dominant accumulating coordinate is sufficient.	independent measurements of v_xeno_, τ_eff_ and *X*_crit_ with other control variables fixed; predicted M regime and, for *M* > 1, comparison of predicted t_crit_ with observed threshold-crossing time. Use a multidimensional transition boundary when required.
Does the resulting structure alter future chemistry?	feedback of retained matter on transport, localization, persistence or flux.	matched rechallenge after removal of the initiating input; transport, mechanical or permeability measurements.

## Where Chemical Foreignness Resides

Foreignness can occur at different points in a reaction sequence, and is not necessarily residing in the molecule itself. It may lie in the starting molecule, in a noncanonical functional group or element, in the product, or in the way otherwise familiar reactions are connected.

In some cases, the individual substrates and reaction types are not themselves unusual, but their combination produces a process with no known biological counterpart. Free-radical vinyl polymerization is a clear example: radicals and carbon–carbon double bonds are common in biology,^18–20^ but sustained addition of vinyl monomers into a growing synthetic polymer chain is not. In this case, the foreign feature is the overall reaction sequence rather than a single substrate.

These categories can overlap. Metabolic oligosaccharide engineering and genetic-code expansion introduce non-native chemical groups through existing biosynthetic pathways. Elemental substitution introduces atoms that are normally absent from a given biomolecule. Fluoroacetate metabolism begins with a foreign substrate. Engineered radical polymerization may involve a foreign monomer, a non-native polymerization mechanism and a product outside the normal biosynthetic repertoire. Conversely, polymerizing a biologically produced monomer such as itaconate^20^ would still constitute foreign chemistry if the polymerization process itself has no biological counterpart.

## From Chemical Possibility to System-Level Realization

What separates a reaction that can occur from one that actually occurs in a cell? Three levels need to be distinguished: chemical possibility, access within the system and observed flux. A reaction may be chemically possible but absent in an intact system because substrate and catalyst do not meet, or because the product is removed faster than it is formed.

Purified-enzyme screens, extracts and broad activity assays can identify reactions that are possible in principle. Screening 667 purified *Escherichia coli* enzymes against intracellular metabolome extracts identified 76 enzymes -about one in nine- with activity toward previously unannotated reactants,^21^ showing that enzyme reactivity extends beyond the annotated metabolic network. However, because membranes, compartmentalization and physiological competition were absent, these measurements do not directly describe cellular flux.

Access depends on whether substrate and catalyst can meet under physiological conditions. Permeability, partitioning, localization and cofactor availability can all affect this. A good substrate *in vitro* may never enter the relevant compartment *in vivo*, or adsorption, phase partitioning or confinement may instead increase its local concentration.

Observed xenochemical flux depends on both formation and removal. Clearance, dilution, export, detoxification, quenching and downstream consumption can all reduce product accumulation. A slowly produced but persistent product may therefore accumulate more than one that is formed rapidly and removed just as quickly. Intact-system flux can also be compared with a matched permissive reference condition to estimate how strongly system organization suppresses or enhances a reaction.

In underground metabolism, enzyme side activities can become relevant under particular physiological conditions and may support alternative metabolic routes. In *E. coli*, for example, different routes to isoleucine are used under aerobic and anaerobic conditions.^8,22,23^ Although these pathways involve native metabolites, they show that the reactions realized by a cell depend on physiological context as well as enzyme repertoire.

State dependence is mechanistic. Cells with identical coding potential can support different xenochemical fluxes when, for example, enzyme abundance, redox state or transport changes. Exposure history can induce later pathway steps, while community composition may add or remove them.^24^ The same compound may therefore be cleared at low dose but accumulate or branch after saturation.

Physical organization can therefore select the realized network (Figure 1 B). Membranes first determine which reactants gain access; confinement or phase partitioning can then enrich them locally, while interfaces and compartment boundaries determine whether intermediates are transferred or lost. Retention at cell surfaces or within extracellular matrices can prolong local exposure, and spatial gradients make these effects heterogeneous across the system. A reaction therefore depends not only on chemical compatibility but on whether the relevant components coexist in space and time. Genetically targeted photocatalysts make this dependence experimentally explicit: organic photocatalytic dyes can be restricted to defined cells and subcellular locations, confining abiotic bond-forming chemistry to selected cellular spaces. Localization can therefore determine not merely reaction efficiency, but where foreign chemical flux is realized.^25^ At a different level of organization, recent theoretical work on multivalent recognition likewise shows how selectivity can emerge from the combined effect of many interactions rather than from a single molecular affinity.^26^

Artificial compartments make these effects easier to isolate experimentally. Coacervate-based protocells can localize enzymes and biomacromolecules and support multistep reactions, while protocell communities can distribute different reactions across separate compartments. These systems are relevant to the xenoreactome when compartmentalization changes catalyst assembly, substrate access or transfer between reaction steps.^27,28^

## Molecular Realization: Transformation, Propagation and Incorporation

Once foreign chemistry enters a biological system, the useful question is how far it propagates. A first conversion may terminate locally, or the resulting chemical feature may survive transfer to another catalyst, compartment or biosynthetic process. Each hand-off can preserve, modify or eliminate it. Covalent adducts can even retain evidence of xenoreactivity after the initiating molecule has disappeared.^29^

Fluoroacetate poisoning provides a simple example. Fluoroacetate is converted to fluoroacetyl-CoA, condensed with oxaloacetate and ultimately converted into a fluorocitrate species that inhibits aconitase. The toxicity arises because a foreign substrate is processed by enzymes of normal carbon metabolism, the classical case of “lethal synthesis”.^30–32^ The relevant network is therefore not a new metabolic pathway, but the route by which native metabolism converts an initially foreign molecule into a toxic product. The same logic applies when a foreign chemical feature is retained rather than destroyed. In metabolic oligosaccharide engineering, a non-native monosaccharide enters the cell, is converted into an activated nucleotide sugar and is then used by the normal glycosylation machinery. The modified sugar can eventually appear in glycans at the cell surface, including in animals and developing zebrafish.^33–35^ The foreign feature therefore survives several biochemical and compartmental steps between uptake and surface display.

Genetic-code expansion follows a similar route in protein synthesis. A noncanonical amino acid must reach the cell, be recognized by an orthogonal aminoacyl-tRNA synthetase, loaded onto tRNA, incorporated by the ribosome and retained in the final protein. Each step places a different constraint on the same foreign chemical feature: it must avoid unwanted reactions while remaining compatible with the machinery needed to carry it forward^36–39^ (Figure 2A). Here, propagation describes the persistence of a foreign chemical feature through successive reactions or transfers before it is removed or reaches a final product. Xenobiological engineering can also alter the routes through which chemical information is processed. Orthogonal ribosome-mRNA pairs establish translation channels with recognition rules distinct from those of the host machinery.^40^ Such orthogonality does not itself imply a new reaction type, but changes which substrates and products can be connected within the system. Genomic integration is distinct again: it can stabilize the genetic instructions supporting a xenochemical capability, but does not by itself establish that the corresponding chemistry is realized or physiologically maintained.^41^ Synthetic auxotrophy provides a stronger functional coupling: engineered essential proteins can make growth dependent on the incorporation of an externally supplied noncanonical amino acid.^42^ At a broader architectural level, genetic firewalls aim to limit exchange with natural biology through incompatibilities in informational chemistry or decoding.^13^ These examples connect xenobiology to xenoreactomic analysis by distinguishing the engineered architecture from the uptake, recognition, incorporation and persistence processes that sustain its chemical operation.

**Figure 2:**
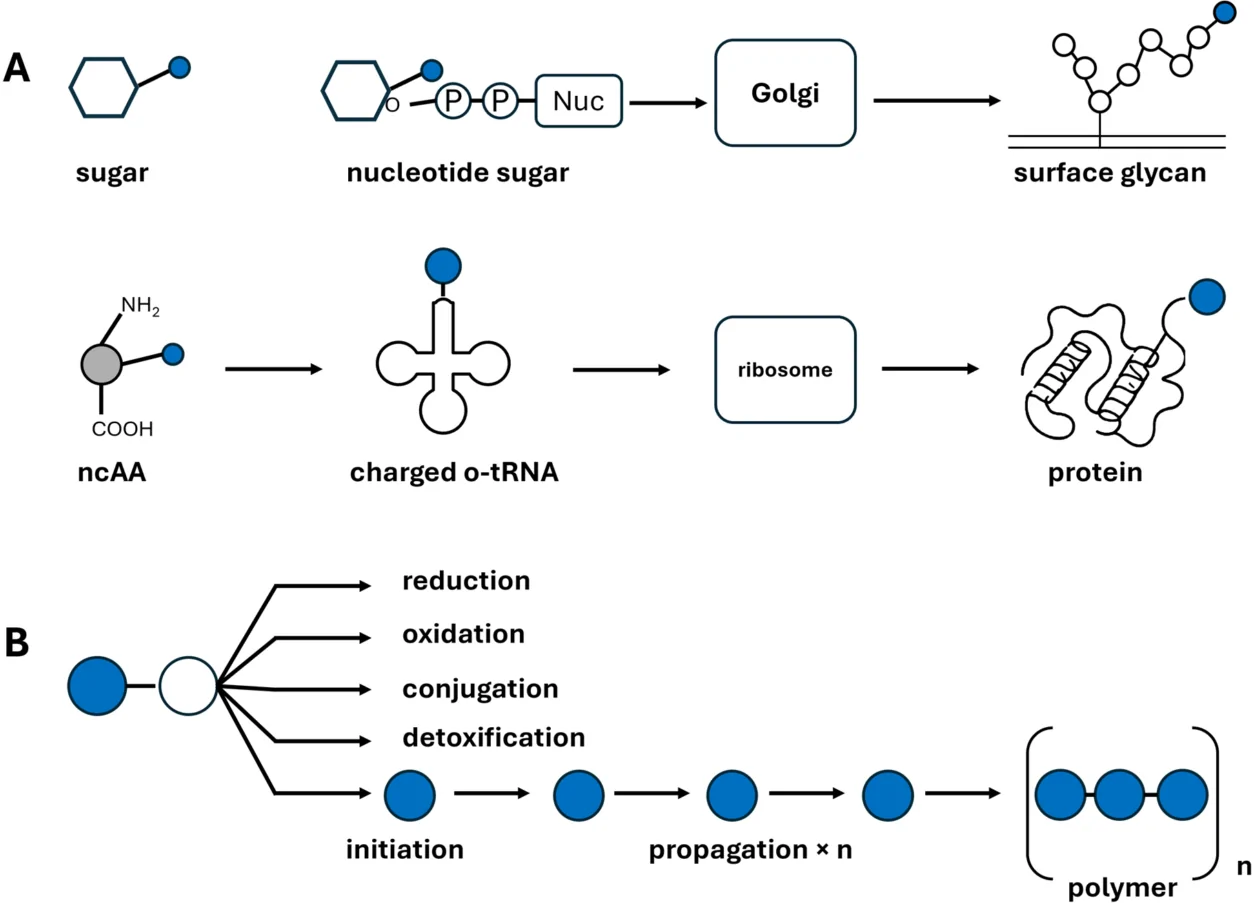
From propagation and incorporation to foreign reaction topology. (A) Metabolic oligosaccharide engineering and genetic-code expansion illustrate how a foreign feature can survive sequential recognition, catalytic transformation and compartmental transfer before becoming displayed in a glycan or retained in a protein. These are examples of foreign chemical content propagated through existing biological construction pathways. (B) Representative biological fates of unsaturated metabolites include reduction, oxidation, conjugation and detoxification; by contrast, sustained free-radical vinyl chain growth organizes repeated addition into macromolecular chain growth. In this case, foreignness resides in the propagation topology rather than necessarily in any individual substrate or elementary reaction.

Foreign chemistry can also involve elements that are rarely or never used in native biomolecules. Boron, for example, can be introduced site-selectively into proteins as boronoalanine by post-translational chemistry. This demonstrates that proteins can accommodate a noncanonical element, although the modification itself does not require an intact cellular system. Telluromethionine provides an even stronger cellular example, as it can replace methionine during recombinant expression in methionine-auxotrophic *E. coli*, coupling uptake of the non-native amino acid to normal translation.^43,44^

Foreign elements can also appear in products that biology does not normally make. Directed evolution of *Rhodothermus marinus* cytochrome c enabled carbon–silicon bond formation, including in *E. coli* whole cells, producing organosilicon compounds through a reaction not known in natural biochemistry.^45^ Silicon can also be incorporated at a larger scale: in cryogenic silicification, mammalian cells organize the formation of an inorganic silica phase rather than a discrete modified metabolite or biomolecule.^46^

In these cases, existing biological machinery carries the foreign feature forward. Radical polymerization differs because the reaction sequence itself has no direct biological counterpart.

## Foreign Reaction Topology: Radical Polymerization

Can the reaction sequence itself be foreign, even when its individual components are not? For instance, radicals and carbon–carbon double bonds are not themselves unusual in biology.^18–20^ What biology does not normally do is couple them into sustained vinyl chain growth. Reactive unsaturated metabolites normally undergo reduction, oxidation, conjugation or covalent addition rather than repeated chain growth. Acrolein, for example, forms biomolecular adducts but does not generate controlled polymers.^19^ A biological monomer does not make the polymerization (Figure 2B).

Such reactions can occur in living cells. Free-radical polymerization of acrylic and methacrylic monomers has been demonstrated inside mammalian cells,^5^ producing intracellular polymers and nanostructures and altering cell behavior.^5^ Related work in *E. coli* showed that intracellular biomolecules can support radical polymerization of several acrylic, acrylamide and methacrylic monomers, extending this chemistry to bacterial cells.^47^ Radical polymerization can also be localized at cell surfaces, thereby modifying the interface through which the cell interacts with its environment. Cytocompatible PET-RAFT has been initiated from chain-transfer agents attached to yeast and mammalian cells,^48^ while surface-displayed horseradish peroxidase has been used to drive bioATRP and bioRAFT from the *S. cerevisiae* cell wall.^49^ Metabolic activity can itself initiate foreign polymerization. Transmembrane redox flux from living bacteria can drive catalyst-free radical polymerization of zwitterionic methacrylates at the cell surface.^50^

Together, these examples show that xenoreactomic coupling need not be intracellular. The cell can therefore shape not only whether polymerization occurs, but where it occurs and what physical relationship the product ultimately has with the cell. The foreign component may therefore lie in the monomer, the polymerization mechanism, the product, or a combination of these. If the polymer persists, it can also change the environment in which subsequent reactions occur. A polymer shell, for example, may affect transport, aggregation or protein presentation and thereby influence later xenochemical activity.^48,49^

## When Xenochemistry Produces Matter

When does foreign chemistry stop being only molecular and begin to produce matter? Of course, most xenoreactomes do not reach this point; material formation is a special case in which a xenochemical product also has the physical capacity to assemble, condense or undergo a phase transition. Some xenoreactomes end with soluble products, while others generate persistent structures. This occurs when production of a xenochemical species exceeds its removal long enough for the product to reach the concentration or density required for assembly or phase transition. Conversely, precipitation or phase separation does not necessarily imply biological integration or function. Natural biomineralization provides a useful physical analogue: fungal oxalate production can drive calcium oxalate precipitation once local conditions favor supersaturation;^51^ enzymatic polymerization of poly­(dimethylaminoethyl methacrylate) can similarly generate enough polymer to trigger complex coacervation with ATP;^52^ in artificial vesicles, surface-confined polyaniline synthesis has been coupled to membrane growth and division.^53,54^ In each case, molecular production changes the physical state of the system once sufficient product accumulates.

For this subset of xenoreactomes, whether materialization occurs depends on whether production can outpace removal long enough to reach the relevant threshold. Consider a xenochemical species *X* produced at a rate *v*_xeno_. Before materialization, *X* is also removed through downstream reactions, repair, export, dilution or other loss processes. If these losses are represented by an effective persistence time τ_eff_, its accumulation can be written as

1 $$\frac{\mathrm{dX}}{\mathrm{dt}}=v_{\mathrm{xeno}}-\frac{X}{\tau_{\mathrm{eff}}}$$

Here, *X* can represent a local concentration, surface density or volumetric density, depending on the system. The effective persistence time τ_eff_ summarizes the loss processes acting before materialization occurs. Under constant production, the corresponding steady-state level is

2 $$X_{\mathrm{ss}}=v_{\mathrm{xeno}}\tau_{\mathrm{eff}}$$

For a purely molecular xenoreactome, this quantity simply describes the balance between production and removal. If the same product can undergo assembly or a phase transition, its steady-state level can instead be compared with an experimentally defined threshold *X*_crit_. We therefore define the materialization number

3 $$M=\frac{v_{\mathrm{xeno}}\tau_{\mathrm{eff}}}{X_{\mathrm{crit}}}=\frac{X_{\mathrm{ss}}}{X_{\mathrm{crit}}}$$

For *M* < 1, the steady state remains below the threshold; *M* = 1 is the asymptotic boundary, whereas *M* > 1 allows threshold crossing in finite time.

Threshold reachability does not, however, mean that the transition will occur within a finite experiment. For X(0) = 0, the time-dependent solution is

4 $$\frac{X\left(t\right)}{X_{\mathrm{crit}}}=M\left(1-e^{-t/\tau_{\mathrm{eff}}}\right)$$

For *M* > 1, the predicted threshold-crossing time is therefore

5 $$t_{\mathrm{crit}}=-\tau_{\mathrm{eff}}\ln\left(1-\frac{1}{M}\right)$$

Thus, *M* determines whether the threshold is reachable at steady state, while *t*_crit_ determines whether it should be reached within the experimental observation window. As *M* approaches 1 from above, the predicted crossing time becomes increasingly long (Figure 3).

**Figure 3:**
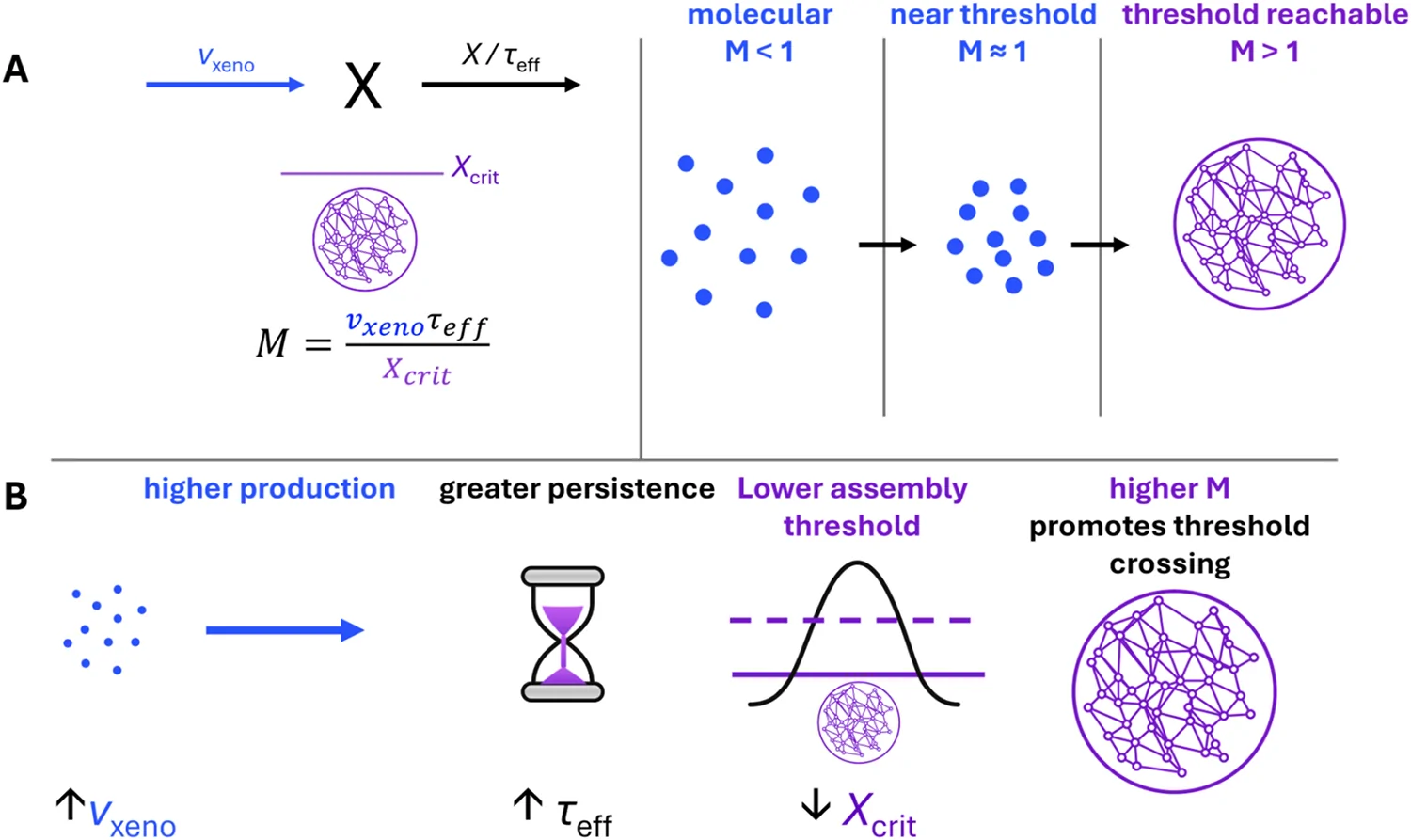
Threshold-dependent material formation in a xenoreactome. (A) A xenochemical species *X* is produced at rate *v*_xeno_ and removed with an effective pretransition persistence time τ_eff_. The materialization number *M* compares its expected steady-state abundance with the experimentally defined materialization threshold. For *M* < 1, the steady state remains below the threshold; *M* = 1 is the asymptotic boundary; for *M* > 1, threshold crossing can occur in finite time, provided *t*_crit_ lies within the observation window. (B) Materialization can be promoted by increasing production, increasing persistence or lowering the relevant transition threshold.

The materialization number is an operational criterion rather than a universal measure of xenoreactome strength. Its parameters should be determined independently before materialization: *v*_xeno_ from product formation, τ_eff_ from pretransition persistence, and *X*_crit_ from the relevant assembly or phase boundary. The reduced model assumes approximately constant production, first-order pretransition loss and one dominant accumulating variable. Systems governed by several coupled variables, strong hysteresis or stochastic nucleation require a more detailed description.

Artificial cells are particularly useful for testing this relation because permeability, volume, spatial organization and product sinks can be varied independently while keeping the underlying reaction chemistry fixed.^27,55,56^

## Realizing New-to-Nature Chemistry in Cells

What changes when a new-to-nature reaction moves from a test tube into a living system? The catalyst is no longer the whole experiment. A reaction must gain access to its substrates, operate in the relevant cellular context and produce intermediates that persist long enough to be carried forward.

Non-native transition-metal catalysis provides clear examples. Transition-metal reactions including palladium-mediated chemistry, gold and ruthenium catalysis and olefin metathesis have all been demonstrated in living cells.^4,57–59^ The catalyst provides the new reaction; the cell decides whether that reaction has somewhere to go. Substrate access, product persistence and downstream metabolism determine whether it remains an isolated event or becomes part of a larger chemical sequence. In parallel, expression in *Pseudomonas putida* of a fluorinase enabled formation of 5′-fluoro-5′-deoxyadenosine and downstream fluorometabolites.^3^ Achieving this chemistry required more than the C–F bond-forming enzyme: the researchers also had to engineer fluoride uptake and tolerance, precursor availability and cellular regulation. Recent examples couple native metabolism directly to new-to-nature chemistry: phosphate-driven Lossen rearrangement can rescue PABA auxotrophy,^60^ endogenous H_2_ can fuel Pd-catalyzed hydrogenation,^61^ and biosynthesized precursors can feed P450 nitrene transfer in living cells.^62^ The resulting xenoreactome remains molecular, but its realization depends on the organization and physiology of the host.

Artificial compartments can provide similar control. In DNA protocells, an encapsulated artificial metalloenzyme coupled olefin metathesis to changes in protocell growth, adaptation and fusion.^63^ Here, localization of the catalyst and its products within the compartment links a non-native reaction directly to the behavior of the system. Purified catalysts establish whether a reaction is chemically possible; in vitro cascades test whether several reactions can operate together; intact cells add transport, localization and physiological constraints. The limiting step may therefore shift from catalysis to substrate access, toxicity, cofactor supply, or product clearance as the chemistry moves into a living system.

## How to Measure a Xenoreactome

How can we tell whether system organization actually matters? Intuitively, the necessary experiments would be perturbational. First establish that the reaction occurs; then change a feature of the system while keeping the underlying chemistry as constant as possible. If access or localization is causal, changing it should alter xenochemical flux. If a hand-off is required, interrupting it should remove downstream products while leaving upstream chemistry intact. Time-resolved analysis, isotope tracing and spatial measurements can then identify where that dependence enters the network. Claims of retention or material formation require direct evidence: foreign chemical features should be localized in the resulting biomolecule or structure, polymers should be chemically and molecularly characterized, and phase transitions should be distinguished from nonspecific aggregation or precipitation. Material-forming systems provide a separate quantitative test. Independently varying *v*_xeno_ and τ_eff_ while holding *X*_crit_ and the other relevant control variables fixed, should predict the threshold regime through M and the finite-time crossing through *t*_crit_. Localization can be tested just as directly by comparing surface-displayed and soluble versions of the same catalyst at matched bulk activity. If both produce the same local product accumulation and cell-associated effects, localization is not a determining component of that xenoreactome.

## Outlook

The central shift introduced by the xenoreactome is from asking what a catalyst can do to asking what chemistry a system can sustain. A weak reaction may become consequential when localization or retention allows its products to accumulate or reach a downstream partner; an efficient catalyst may remain irrelevant when access fails or its products are rapidly removed. Transport, compartmentalization and persistence therefore become variables with which foreign chemistry can be designed, rather than background conditions around the reaction.

The boundary between foreign and native chemistry is fluid. In *Pseudomonas* sp. strain ADP, plasmid-borne *atz* genes support atrazine degradation,^64^ while *Ideonella sakaiensis* couples PET depolymerization to assimilation of the resulting products.^65^ Adaptation can otherwise stabilize foreign chemistry by reshaping the physiological context that sustains it, rather than the xenochemical transformation itself.^66^ Reactions that begin as rare side activities can therefore become integrated through changes in expression, regulation, transport or enzyme specificity.^1,6,8^ These changes define a possible, but not obligatory, path from chemical realization through physiological accommodation to stable integration and evolutionary stabilization. Selection can act on the surrounding transport, regulatory and physiological architecture as well as on the xenochemical transformation itself. Most foreign reactions will never reach that point, but only chemistry realized in the intact system can become exposed to selection. A next step is to compare the same foreign chemistry in systems that differ in transport, localization and clearance. Defining the reference repertoire, physiological state and observation window would help make these comparisons meaningful. Measuring substrate access, product flux and residence time could show what limits the chemistry: catalysis, delivery, transfer between reaction steps or product loss. Exposure and withdrawal experiments could then reveal whether the chemistry stops with the input or leaves lasting changes in the system. Together, these tests would show whether the framework can predict outcomes across conditions.

For material-forming xenoreactomes, the question is when product accumulation produces a new physical state, and how that state affects later reactions. Artificial cells allow permeability, volume and product removal to be varied under controlled conditions. Living cells can reveal how these effects change in the presence of competing physiological processes. Such experiments could guide the design of systems that sustain foreign chemistry, retain its products or turn them into materials.

The framework should therefore be judged by experiments that can falsify these dependencies. If changing a proposed organizational constraint leaves xenochemical flux unchanged, that constraint is not part of the realized network. Breaking a required hand-off should eliminate downstream chemistry while preserving the upstream reaction. In material-forming systems, independently measured production, persistence and threshold should predict whether (and on what time scale) the transition is reached. The broader question is no longer only what foreign reactions biology can tolerate, but what kind of chemistry an organized biological system can be made to realize, and how far that chemistry can reshape the matter from which the system is built.
